# Supplementary material for: Nurses’ Perceptions of the Quality of Procedural Sedation in Children Comparing Different Pharmacological Regimens
Source: Children (Basel). 2022 Jul 18;9(7):1068. doi: 10.3390/children9071068 (PMC9315654; doi:10.3390/children9071068)
Supplement: Supplementary file 1 [file children-09-01068-s001.zip › children-1794607-supplementary.pdf]

Supplementary material 1

Survey on pediatric procedural sedation

1. Indicate in which department you work

2. For how many years have you been working in the field of pediatric sedation?

3. In the department where you work, how many sedations / recoveries from sedation per month do you normally attend?

- <10
- 10-20
- 21-30
- >30

4. For each of the following drugs, please rate your level of satisfaction for the overall quality of sedation? (0 = very unsatisfied, 10 = very satisfied; NA = not applicable).

- Propofol 0 1 2 3 4 5 6 7 8 9 10 NA
- Ketamine 0 1 2 3 4 5 6 7 8 9 10 NA
- Midazolam 0 1 2 3 4 5 6 7 8 9 10 NA
- Dexmedetomidine 0 1 2 3 4 5 6 7 8 9 10 NA

5. For each of the following drugs, please select the adverse effects you witness most frequently during or after children's sedation (0 = sleepiness, 1 = irritability, 2 = hyperactivity / restlessness, 3 = hallucinations, 4 = unsteadiness, 5 = headache, 6 = dizziness, 7 = alterations in appetite, 8 = nausea / vomiting, 9 = respiratory distress, 10 = none; NA = not applicable). More than one answer is possible.

- Propofol 0 1 2 3 4 5 6 7 8 9 10 NA
- Ketamine 0 1 2 3 4 5 6 7 8 9 10 NA
- Midazolam 0 1 2 3 4 5 6 7 8 9 10 NA
- Dexmedetomidine 0 1 2 3 4 5 6 7 8 9 10 NA

6. For each of the following adverse events, please rate how much their presence affects your satisfaction. (1= None influence, 2 = Little influence, 3 = Medium influence, 4= Large influence).

   - Sleepiness 0 1 2 3 4
   - Irritability 0 1 2 3 4
   - Hyperactivity / restlessness 0 1 2 3 4
   - Hallucinations 0 1 2 3 4
   - Unsteadiness 0 1 2 3 4
   - Headache 0 1 2 3 4
   - Dizziness 0 1 2 3 4
   - Alterations in appetite 0 1 2 3 4
   - Nausea / vomiting 0 1 2 3 4
   - Respiratory distress 0 1 2 3 4

7. Please indicate for which of the following drugs you feel the administration of a rescue drug (eg antiemetic drug) is more often required during or after children's sedation? More than one answer is possible.

- Propofol
- Ketamine
- Midazolam
- Dexmedetomidine

8. For each of the following drugs, please rate your perceived level of safety during or after children's sedation (0 = not safe, 10 = very safe; N / A = not applicable)?

- Propofol 0 1 2 3 4 5 6 7 8 9 10 NA
- Ketamine 0 1 2 3 4 5 6 7 8 9 10 NA
- Midazolam 0 1 2 3 4 5 6 7 8 9 10 NA
- Dexmedetomidine 0 1 2 3 4 5 6 7 8 9 10 NA

If you do not feel safe, please specify why

9. Which route of administration would you prefer to be used for pediatric sedation?

- Oral + intranasal route
- Intranasal route
- Intravenous route

- Intramuscular route

10. Which drug would you prefer to be used for pediatric sedation?

- Propofol
- Ketamine
- Midazolam
- Dexmedetomidine

11. How useful do you rate the use of non-pharmacological techniques when performing painless / minimally painful procedures in children (e.g. peripheral line placement)?

- Useless
- Mildly useful
- Useful
- Very useful

12. Regardless of the type of sedation, in which scenarios would you prefer to have a peripheral venous access in place?

- Always
- Always in "difficult" children (e.g., children with genetic syndromes, autism, cognitive impairment)
- Never

13. Please indicate which of the following aspects of sedation affect your satisfaction more deeply. More than one answer is possible.

- Type of drug
- Route of administration
- Presence of side effects during sedation
- Presence of side effects during recovery
- Quality of recovery
- Awaking times
- Need for rescue drugs
- Nursing staff availability
- Parental satisfaction
- Children's satisfaction

- Other

What would you like to be changed from the current standards of pediatric procedural sedation in your operating unit? Optional question.

If your child needed to undergo a procedural sedation, which drug(s) and route of administration would you like to be used? Optional question.
